# Multicore Assemblies from Three-Component Linear Homo-Copolymer Systems: A Coarse-Grained Modeling Study

**DOI:** 10.3390/polym13132193

**Published:** 2021-06-30

**Authors:** Sousa Javan Nikkhah, Elsi Turunen, Anneli Lepo, Tapio Ala-Nissila, Maria Sammalkorpi

**Affiliations:** 1 Department of Chemistry and Materials Science, School of Chemical Engineering, Aalto University, P.O. Box 16100, FI-00076 Aalto, Finland; 2 Department of Physics, Bernal Institute, University of Limerick, V94T9PX Limerick, Ireland; 3 R&D and Technology, Kemira Oyj, P.O. Box 44, FI-02271 Espoo, Finland; elsi.turunen@kemira.com (E.T.); anneli.lepo@kemira.com (A.L.); 4 QTF Centre of Excellence, Department of Applied Physics, Aalto University, FI-00076 Aalto, Finland; tapio.ala-nissila@aalto.fi; 5 Centre for Interdisciplinary Mathematical Modelling and Department of Mathematical Sciences, Loughborough University, Loughborough, Leicestershire LE11 3TU, UK; 6 Department of Bioproducts and Biosystems, School of Chemical Engineering, Aalto University, P.O. Box 16100, FI-00076 Aalto, Finland

**Keywords:** polymer self-assembly, multicore assembly, multicompartment assembly, multicore micelle, multicompartment micelle, block copolymer, dissipative particle dynamics

## Abstract

Multicore polymer micelles and aggregates are assemblies that contain several cores. The dual-length-scale compartmentalized solvophobic–solvophilic molecular environment makes them useful for, e.g., advanced drug delivery, high-precision synthesis platforms, confined catalysis, and sensor device applications. However, designing and regulating polymer systems that self-assemble to such morphologies remains a challenge. Using dissipative particle dynamics (DPD) simulations, we demonstrate how simple, three-component linear polymer systems consisting of free solvophilic and solvophobic homopolymers, and di-block copolymers, can self-assemble in solution to form well-defined multicore assemblies. We examine the polymer property range over which multicore assemblies can be expected and how the assemblies can be tuned both in terms of their morphology and structure. For a fixed degree of polymerization, a certain level of hydrophobicity is required for the solvophobic component to lead to formation of multicore assemblies. Additionally, the transition from single-core to multicore requires a relatively high solvophobicity difference between the solvophilic and solvophobic polymer components. Furthermore, if the solvophilic polymer is replaced by a solvophobic species, well-defined multicore–multicompartment aggregates can be obtained. The findings provide guidelines for multicore assemblies’ formation from simple three-component systems and how to control polymer particle morphology and structure.

## 1. Introduction

Multicore polymer assemblies, i.e., polymer aggregates consisting of multiple microphase-segregated cores and in the case of micelles, a solvophilic corona, are functional self-assembling materials with important applications in, e.g., water purification, catalysis, pharmacology, electronics, and oil recovery [1,2,3]. The high tunability of the chemistry, size, and distribution of the solvophobic cores of the multicore assemblies make them unique nanoscale capsules that can be loaded with chemical reagents or catalysts, making them nanoreactors [4]. Such polymeric capsule nanoreactors are ubiquitous, for example, in emulsion polymerization [4,5] and confined catalysis [6,7], but also as artificial organelles in synthetic biology and therapeutic applications [8,9,10].

Furthermore, multicore–multicompartment assemblies and micelles, which differ from the basic multicore micelles by having multiple levels of compartmentalization, are used for encapsulating incompatible catalysts that should not interact with each other in cascade reactions [6,11,12,13,14,15]. In drug delivery, these assemblies provide isolated environments for combinations of drugs with different chemical characteristics and release profiles. Such multi-drug carriers are promising to treat cancer tumors as they enable treatment aimed at overcoming tumor heterogeneity, reduce chemoresistance, and offer more desirable synergistic anticancer efficacy without overlapping toxicity [16,17]. Furthermore, the controllability of both the core chemistries and sizes make these assemblies highly versatile. These reasons make finding easy and economic methods to produce multicore and multicompartment assemblies, as well as guidelines for their assembly, immensely important. Here, we present a study towards facilitating systematic design of assemblies in which the concentrated phase spontaneously separates to form segregated droplets in a cost-efficient, simple block-copolymer framework. We refer to aggregates with this droplet-like internal phase-separation as multicore assemblies.

In recent years, multicore or multicore–multicompartment assemblies, in particular micelles, have successfully been produced by various approaches based on self-assembly of triblock copolymers that include a hydrophilic and two reciprocally incompatible hydrophobic blocks [18,19,20]. Kubowicz et al. [21] employed linear ABC triblock copolymers of poly(4-methyl-4-(4-vinylbenzyl)morpholin-4-ium chloride)-block-polystyrene-block-poly(pentafluorophenyl 4-vinylbenzyl ether) (PVBM-*b*-PS-*b*-PVBFP) in aqueous medium to form spherical multicompartment aggregates with distinct internal structure regions. Multicore micelles of the triblock poly-(ethylene oxide)-*b*-polystyrene-*b*-poly (acrylic acid) were produced by Duxin et al. [22]. The morphology of the self-assembled structures is significantly influenced by the polymeric building block molecular characteristics, such as composition, but also the number, length, and sequence of segments. Thunemann et al. [23] examined the assembly of dilute aqueous solution of a linear ABCBA penta-block copolymer and obtained spherical and cylindrical multicompartment micelles with two cores that each consisted of dominantly different block species. Another influential control factor of the final aggregate morphology is the architecture of triblock copolymers: comb, miktoarm, and dendritic architectures have so far been considered particularly suitable for the formation of true multicompartment structures [24]. Li et al. [25] synthesized a series of ABC miktoarm star triblock copolymers of m-[poly (ethyl ethylene)] [poly (ethylene oxide] [poly (perfluoropropylene oxide)] (m-EOF) and studied the assembly of these in dilute aqueous solution to obtain distinctly two-compartment micelles. Iatridi et al. [26] reported multicore micelles using multi-arm, star-shaped ter-polymer containing nine polystyrene arms and nine poly (2-vinyl-pyridine)-*b*-poly (acrylic acid) arms. Ueda et al. [27] reported multicore micelles of sodium maleate and dodecyl vinyl ether copolymers, and they also observed a single-core to multicore micelle transition in their system.

Despite all the successful demonstrations of assembly and usefulness of the multicore and multicompartment micelles, at a more general level, the guidelines for the multicore self-assembly process in polymeric systems and the factors leading to multicore assembly remain unresolved. Additionally, at the microscopic level, spontaneous multicore assembly and control of the assemblies remain open topics. Furthermore, the polymer systems for which multicore or multicompartment assembly morphologies have been demonstrated are relatively complex.

Computer simulations provide a powerful tool to extract molecular level interdependencies that may be difficult to extract empirically. Copolymer micellization and aggregation in solution has been examined with various computational approaches. These include self-consistent field theory (SCFT) [28,29,30,31,32,33,34], Monte Carlo simulations [35,36,37,38], Brownian dynamics [39,40,41], dissipative particle dynamics (DPD) simulations [42,43,44,45,46], and at more limited length and time scales, coarse-grained and atomistic molecular dynamics simulations [47,48,49,50]. As multicore self-assembly has both high practical significance and interest from theory and computational points of view, different theoretical and computational works have been devoted to multicore aggregation. On the theory side, Wang et al. [34], via a self-consistent field theory approach, have studied multicore micelle formation from linear ABC tri-block ter-polymers containing a solvophilic midblock and two mutually incompatible solvophobic head groups. In another study, Wengenmayr et al. [51], via a mean-field model, observed the splitting of unimolecular single-core micelles of dendritic-linear copolymers to a multicore structure, with increasing dendrimer generation and decreasing solvent selectivity. On the computational side, DPD simulations which employ particle-based, mesoscale level modeling of the polymer chains and their assembly and dynamics, have turned out to be an adaptable and reasonably successful tool for examining the formation and assembly responses of polymeric micelles [46,52,53,54]. In particular, multicore and multicompartment micelles have been studied via DPD by Zhao et al. [3], who investigated the formation of multicompartment micelles from a dilute aqueous solution of the mixture of di-block copolymer poly (ethyl ethylene)-block-poly (ethylene oxide) (PEE-*b*-PEO) and homopolymer poly(propylene oxide) (PPO). Chen et al. [54] also employed DPD simulations to examine the multicomponent–multicore micelle formation from two kinds of star-shaped copolymers and the degradation of multicomponent–multicore micelles. Chen et al. [55], via DPD, examined the self-assembly of π-shaped copolymers that had rigid backbones and two flexible side chains in a selective solvent. By varying the model parameters, such as the concentration of the copolymer, the nature of the solvent, and the repulsive interactions between the two coiled side chains and the rigid backbone block, they observed a variety of nanostructures, including interacting micelles, multicore micelles, nanocages, and nano-tires. Callaway et al. [2] studied the self-assembly of a lipophilic−hydrophilic−fluorophilic triblock copolymer system that formed non-spheroidal micelles with multicores of both lipophilic and fluorophilic species.

To find a more simple and economical approach of forming multicore assemblies, we present here a study where an amphiphilic di-block copolymer and two linear homopolymers, one weakly and the other strongly solvophobic, are mixed in a solvent. Since the two homopolymers have a significant difference in their level of solvophobicity and the weakly solvophobic species is relatively compatible with the solvent, we denote the weakly and strongly solvophobic homopolymers as solvophilic (solvophile) and solvophobic (solvophobe), respectively. Such a setup is easily attainable in industrial scale application processes and provides many readily accessible tuning parameters, for example, choice of the polymers, their concentration, degrees of polymerization, and system composition. To reduce the number of variables in the system, the di-block components are considered identical in chemistry to the free solvophile and solvophobe. Here, we show the polymer property range that leads to multicore assemblies, via DPD simulations of this model system. We map the aggregation dependencies and report the range of polymer system parameters over which multicore assemblies form for a fixed degree of polymerization. Furthermore, well-defined multicompartment aggregates can form if the solvophilic polymer is replaced by a more solvophobic species. 

## 2. Materials and Methods

### 2.1. Simulation Details

Polymer particle formation and aggregation was examined via DPD [56]. It is a mesoscale simulation method in which a group of atoms comprise beads that capture the collective interactions of the encompassed atoms and groups. In the DPD approach, the total force exerted on the *i*th bead by all the other beads in the system is provided as:

(1) $${\vec{F}}_{i}=\underset{i\neq j}{\sum }\left({\vec{F}}_{ij}^{C}+{\vec{F}}_{ij}^{D}+{\vec{F}}_{ij}^{R}+{\vec{F}}_{ij}^{S}\right)$$

Here, the four force components correspond to a conservative force ${\vec{F}}_{ij}^{C}=a_{ij}\omega^{C}\left(r_{ij}\right){\vec{e}}_{ij}$, a dissipative force ${\vec{F}}_{ij}^{D}=-\gamma \omega^{D}\left(r_{ij}\right)\left({\vec{\nu }}_{ij}.{\vec{e}}_{ij}\right){\vec{e}}_{ij}$ represents hydrodynamic drag, a random force ${\vec{F}}_{ij}^{R}=-\sigma \omega^{R}\left({\vec{r}}_{ij}\right)\xi_{ij}\Delta t^{-\frac{1}{2}}{\vec{e}}_{ij}\;$corresponds to thermal fluctuations and has Gaussian distribution, and a harmonic spring force ${\vec{F}}_{ij}{}^{s}=C{\vec{r}}_{ij}$ acts between the polymer beads. The spring force is specific to the DPD setup for a polymer system and exists only for neighboring beads in the chain. 

In Equation (1), the total force is summed over all particles i≠j, within a cut-off radius $R_{c}$. Here, ${\vec{r}}_{ij}={\vec{r}}_{i}-{\vec{r}}_{j}$, and $r_{ij}=\left|{\vec{r}}_{ij}\right|,\;{\vec{e}}_{ij}={\vec{r}}_{ij}/r_{ij}\;$, where ${\vec{r}}_{i}\;$and ${\vec{r}}_{j}\;$are the positions of bead *i* and bead *j*, respectively. ${\vec{\nu }}_{ij}={\vec{\nu }}_{i}-{\vec{\nu }}_{j}$, where ${\vec{\nu }}_{i}\;$and ${\vec{\nu }}_{i}$ are the velocities of beads *i* and *j*, respectively. The constant *a_ij_* describes the maximum repulsion between interacting beads. γ and σ are the friction coefficient and noise amplitude, respectively. $\xi_{ij\;}$is a random number with zero mean and unit variance, chosen independently for each interacting pair of beads at each time step Δt, while $\omega^{C}$, $\omega^{D}$, and $\omega^{R}\;$are three weight functions for conservative, dissipative, and random forces, respectively. For the conservative force, we chose $\omega^{C}\left(r_{ij}\right)=1-r_{ij}/R_{c}$ for $r_{ij}<R_{c\;}$and $\omega^{C}\left(r_{ij}\right)=0\;$for$\;r_{ij}\geq R_{c}$. The choice of $\omega^{D}\left(r_{ij}\right)$ and $\omega^{R}\left(r_{ij}\right)\;$is connected via the relation $\omega^{D}={\left[\omega^{R}\left(r_{ij}\right)\right]}^{2}$, $\sigma^{2}=2\gamma k_{B}T$, which comes from the fluctuation-dissipation theorem. Here, we chose a simple form of $\omega^{D}\;$and $\omega^{R}\;$following Groot and Warren [57]: $\omega^{D}\left(r_{ij}\right)={\left[\omega^{R}\left(r_{ij}\right)\right]}^{2}={\left(1-\frac{r_{ij}}{R_{C}}\right)}^{2}$ for $r_{ij}<R_{c\;}$and $\omega^{D}\left(r\right)={\left[\omega^{R}\left(r\right)\right]}^{2}=0$ for $r_{ij}\geq R_{c}.$

The equations of motion in the DPD simulations are integrated using a modified version of the velocity-Verlet algorithm. For simplicity, the DPD simulations employ reduced units such that the cut-off radius $R_{c}$, the bead mass *m*, and $k_{B}T$ specify the unit magnitude for the distance, mass, and energy units, i.e., $R_{c}=m=k_{B}T=1$.

For the like-like-repulsion parameters, *a_ii_*, in Equation (1), Groot and Warren derived an expression $a_{ii}\equiv a_{jj}=\frac{75}{\rho }\;,\;$based on the equation of state for the soft repulsive DPD fluid and the compressibility of bulk water. Here, *ρ* is the number density in the reduced units. For *p* = 3, $a_{ii}$ = 25, which corresponds to the compressibility of water. They also suggested the following linear relation between *a_ij_* and the Flory–Huggins χ parameter at *p* = 3:

(2) $$a_{ij}-a_{ii}\approx 3.27\;\chi_{ij}$$

The harmonic spring force constant *C* was set to be 4.0 [3] to keep the polymer beads connected. 

The bead interaction parameters, *a_ij_*, are presented in Table 1. Here, we take the interaction parameters between the same types of A, S, and W beads to be *a*_WW_ = *a*_SS_ = *a*_AA_ = 25, reflecting the compressibility of water. This value also describes unbiased miscibility between the components, i.e., χ = 0 in the Flory–Huggins model. We set the interaction parameter between the solvophilic block S and the solvent W to *a*_SW_ = 30 (χ = 1.53), as this resides between *a_ij_* = 26.64 that emulates the θ-state (χ = 0.5) and the critical value for hydrophobicity *a_ij_* = 32 [58]. The value of the interaction parameter between A and S is selected as *a*_AS_ = 72 (χ = 14.37). This implies that A and S components are mutually incompatible. The interaction parameter between A and solvent bead W is selected as *a*_AW_ = 115. This value models extremely unfavorable interactions corresponding to the Flory–Huggins parameter χ = 27.72. 

Throughout, we consider a system with 20% of polymeric phase concentration. The basic system is composed of A_19_ (4%), A_1_-*b*-S_6_ (13%), and S_6_ (3%) in solvent medium. As our target is to provide a general perspective to the aggregation behavior of the system chosen, the parameters employed do not rise from a bottom-up coarse-graining of a particular chemical system. However, the bead-spring chain models were chosen to be in the range of oligomers; generally, in a bottom-up coarse-graining study with DPD methodology, each bead-spring model chain can be mapped on a real polymer chain with a degree of polymerization in a range of oligomers to polymers, depending on the degree of coarse-graining. 

The DPD simulations were carried out in a fully periodic cubic simulation box of size 40*R*_c_ × 40*R*_c_ × 40*R*_c_. Assessment of the simulation box size was performed by reproducing the multicore morphologies in a significantly larger system of 100*R*_c_ × 100*R*_c_ × 100*R*_c_, see Supplementary Materials for details. All simulations were carried out using the LAMMPS (large-scale atomic-molecular massively parallel simulator) package [59]. The DPD simulations were performed in the *NVT* ensemble (*k*_B_*T* = 1). The equations of motion were integrated using the modified velocity-Verlet algorithm, with *λ* = 0.65 and a time step Δt=0.05τ. The total simulation times varied between 5 × 10^5^ and 5 × 10^6^ steps. 

### 2.2. Characterization of Self-Assembled Structures 

Classification to aggregates was performed based on a separation distance criterion [60,61]: any bead, or set of beads, within a cut-off distance 1.5*R_c_* from each other are part of the same aggregate. The cut-off distance was determined via visual analysis. The classification scheme is not particularly sensitive to the precise value of the cut-off distance, and the same value was used for all systems. 

The aggregate sizes were characterized by calculating the average particle number (${\bar{X}}_{n}$) and the weight average (${\bar{X}}_{w}$), as:

(3) $${\bar{X}}_{n}=\frac{\sum_{i}X_{i}N_{i}}{\sum N_{i}}$$

(4) $${\bar{X}}_{w}=\frac{\sum_{i}X_{i}^{2}N_{i}}{\sum_{i}X_{i}N_{i}}$$

where *N_i_* is the number of aggregates containing *X_i_* beads. Equilibration of the simulation system was assessed via monitoring the aggregate size distributions, and equilibrium state was deemed when the average aggregate size stabilizes at a constant level. Polydispersity was evaluated via the polydispersity index:

(5) $$P=\frac{{\bar{X}}_{w}}{{\bar{X}}_{n}}$$

Notably, *P* = 1 corresponds to a monodisperse system with identical core sizes. The larger the deviation from unity, the more polydisperse the cores in the assemblies are. 

The internal structure of the aggregates and phase separation in them was quantified via defining an average order parameter, *φ,* as:

(6) $$\varphi =\left(\overset{N}{\underset{i=1}{\sum }}\left|\;\varphi_{Ai}-\varphi_{Bi}\right|\right)/N$$

where the summation is over *N* slices of equal thickness along the *xy*-plane, and in each slice, the volume fractions of beads A and B are *φ_Ai_* and *φ_Bi_*, respectively.

Shape of the aggregates was assessed by determining their deviation from the spherical shape via asphericity, δ, calculated as:

(7) $$\delta =\frac{{\left(\lambda_{1}^{2}-\lambda_{2}^{2}\right)}^{2}+{\left(\lambda_{1}^{2}-\lambda_{3}^{2}\right)}^{2}+{\left(\lambda_{3}^{2}-\lambda_{2}^{2}\right)}^{2}}{2{\left(\lambda_{1}^{2}+\lambda_{2}^{2}+\lambda_{3}^{2}\right)}^{2}}$$

where *λ*_1_^2^, *λ*_2_^2^, and *λ*_3_^2^ are the principal moments of the gyration tensor and *λ*_l_ > *λ*_2_ > *λ*_3_. The value of *δ* is positive, except for a perfect sphere, where it is precisely zero.

## 3. Results

**Figure 1:**
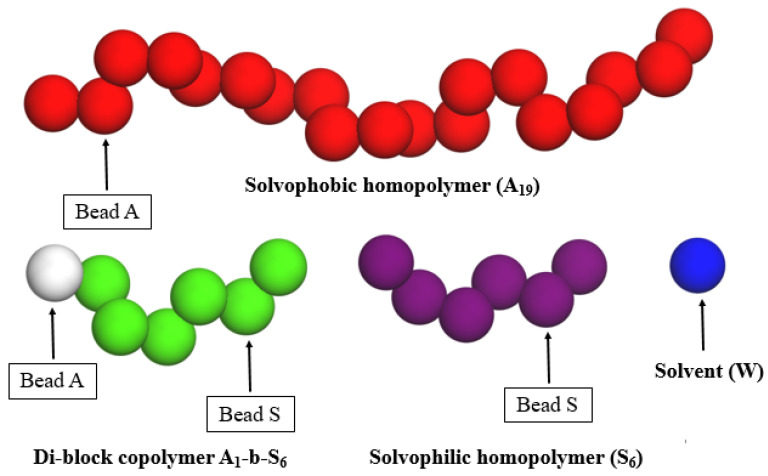
Schematic illustration of the solvophobic and solvophilic homopolymer, block copolymer, and solvent. Different color codes for beads A and S in the copolymer (white and green) and homopolymer (red and purple) chains are to clarify the chain locations in the assemblies in the simulations. Despite the color coding, beads A and S are chemically identical in both polymers.

**Figure 2:**
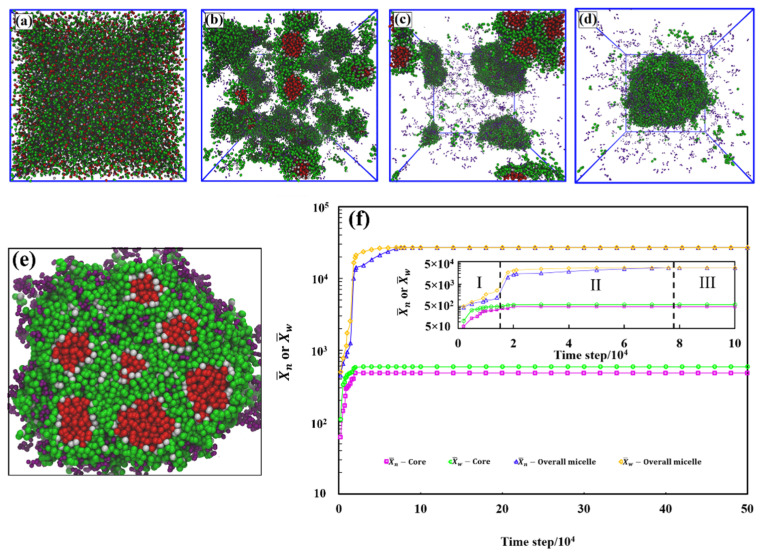
Spontaneous aggregation in a system consisting of A_19_ (4%), A_1_-*b*-S_6_ (13%), and S_6_ (3%) in a simulation system of size (40R_c_)^3^. An initially random mixture (panel **a**) evolves in 5.0 × 10^5^ steps into a spherical multicore assembly (panel **d**). The snapshots correspond to simulation times (**a**) zero, (**b**) 4 × 10^3^, (**c**) 1.3 × 10^5^, and (**d**) 5.0 × 10^5^ steps. Panel (**e**) shows the cross-section of the multicore assembly formed and (**f**) the dependence of the average particle number and the weight average on time. The inset shows magnification of panel (**f**) at early times, where the different stages of evolution, I–III (stage (I) core and micelle formation, stage (II) small micelle aggregation, and stage (III) equilibration) are discussed in the text. Simulation data standard deviations after equilibration are smaller than the symbol size, and hence no error bars are explicitly presented. The polymer bead colors follow Figure 1. Solvent is omitted in the visualizations for clarity.

The DPD simulations comprise a mixture of a linear solvophobic polymer, a linear solvophilic polymer, and a linear amphiphilic di-block copolymer in solvent medium, referred to as A_x_, S_y_, and A_c_-*b*-S_d_, respectively. The model of the system components is shown in Figure 1. The subscripts in each abbreviation refer to the chain length. In all simulation runs, the initially randomly dispersed solvophobic-free chains self-assembled into small micelles very quickly. After this, small micelles gradually formed, and coalesced to form large multicore assemblies. Figure 2 shows the time evolution of the aggregation process corresponding to this self-assembly for a 20% polymer concentration of A_19_ (4%), A_1_-*b*-S_6_ (13%), and S_6_ (3%) in aqueous solutions by simulation snapshots, and the corresponding average sizes of aggregates measured by the average bead number and its weighted average in the aggregates of the system. As an initial step, we extensively tuned the polymer system composition. The final self-assembling structure is highly dependent on the composition of the polymer mixture. This composition was selected as the focus of the work since multicore assemblies readily and stably assembled in this composition and other close-by compositions tested.

The initially formed small micelles are single-core aggregates with double-core corona structure. They are composed of a solvophobic spherical core of A_19_, a layer of solvophobic block of the copolymer as the first corona shell, and both a solvophilic block of copolymer and S_6_ as the second shell. Finally, the small micelles merge with the neighboring micelles to a large spherical aggregate. As shown in Figure 2a–d, the small micelles agglomerate, which is followed by partial merging of their corona regions. However, the small hydrophobic cores remain as separate hydrophobic compartments in the final aggregate. Figure 2e shows a zoomed-in cross-section of the final multicore configuration. Additional time evolution snapshots and a visualization of the aggregate internal structure are provided in the Supplementary Materials Figure S1. Moreover, comparison with the assembly response in the system with the box size of 100*R*_c_ × 100*R*_c_ × 100*R*_c_ (see the Supplementary Materials Figure S2) shows that the simulation box size influences only the aggregate size but not the multicore structure.

Due to the simulation box size dependence of the particle-like assembly size in the simulations, we note that a more general interpretation of the multicore assembly response is a concentrated phase with phase-separation into multiple droplets. In the case of a finite sized aggregate, this would refer to the formation of a concentrated phase droplet containing several small droplets that differ in their polymer composition from the surroundings. 

The time evolution of the aggregate sizes in terms of ${\bar{X}}_{n}\;$and ${\bar{X}}_{w}\;$for both the solvophobic cores and total aggregates in Figure 2f shows three stages of evolution. In the first stage (I), small single-core micelles, with the cores and double-corona composed A_19_ and A_1_-*b*-S_6_ respectively, are formed, in stage (II), the small micelles aggregate to form the multicore aggregate, and stage (III) corresponds to equilibrium. In stage (I), ${\bar{X}}_{n}$ and ${\bar{X}}_{w}$ increase for both the A_19_ solvophobic cores and the whole aggregate, and the nucleation of several small aggregates takes place. At the end of stage (I), the small micelles reach their approximate equilibrium morphology and size, but the total aggregate is still evolving both in shape and size. In the second stage (II), ${\bar{X}}_{n}$ and ${\bar{X}}_{w}$ for the solvophobic cores saturate, but the whole aggregate still increases in size. This stage occurs over a longer time period than the initial micelle formation as the system changes slowly by the small micelles coalescing their solvophilic outer coronas. Such events show a sharp increase in ${\bar{X}}_{n}$ and ${\bar{X}}_{w}$ of the whole aggregate in Figure 2. This process may also have started in stage (I). The final stage (III) is equilibrium, in which ${\bar{X}}_{n}$ and ${\bar{X}}_{w}$ for the whole aggregate saturate, too. The different stages are visualized by the snapshots in Figure 2a–d. 

The different formation stages were also assessed via calculating the order parameter both for the solvophobic chains’ phase separation to form the cores and for the polymer components (solvophobic, di-block copolymer, and solvophilic chains) to form overall aggregates. A larger value of order parameter indicates a higher degree of order, or in this case, component segregation [62]. For the overall aggregate, order parameter values of 0.514 ± 0.001 and 0.134 ± 0.001 were obtained for the final aggregates and the solvophobic cores, respectively. The time evolution of the order parameter is presented in the Supplementary Materials Figure S3. The data show the same response as demonstrated via the ${\bar{X}}_{n}$ and ${\bar{X}}_{w}$ datasets in Figure 2, including the different stages of aggregation. To examine the dispersity of the cores and aggregation, we also calculated the polydispersity, *P*, from Equation (5). The polydispersity data presented in Supplementary Material Figure S4 show that the initially forming polydisperse cores become more monodisperse in size during stage (I) and early in stage (II), i.e., the hydrophobic cores still evolve in stage (II). The practical significance of this is that controlling the growth rate in stage (II) provides a key handle for controlling the size polydispersity of the small cores in applications. For this purpose, e.g., the copolymer block lengths, the chemical difference between the blocks and copolymer concentration provide easily accessible means. 

**Figure 3:**
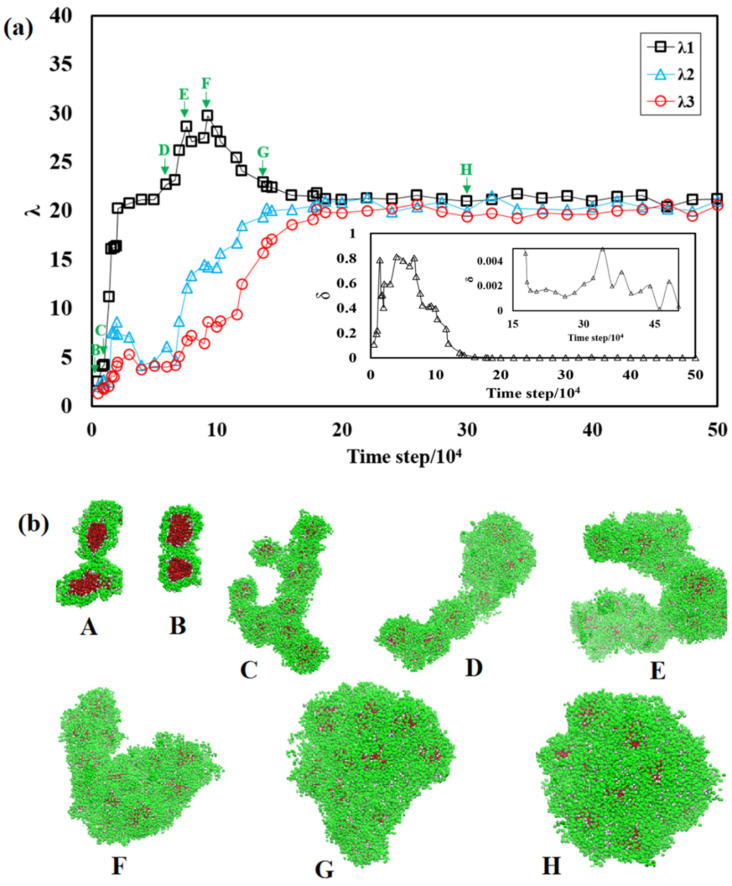
(**a**) The time evolution of λ_l_, λ_2_, and λ_3_ of the largest aggregates at different simulation steps. The embedded figure is the corresponding asphericity, δ. (**b**) The corresponding morphologies of the largest aggregates. Solvent beads are omitted for clarity. The color codes are the same as those in Figure 1.

To assess the shapes of the aggregates and their fluctuations, the time evolution of the principal components of the gyration tensor (λ_l_, λ_2_, and λ_3_) for the largest aggregate in the system are presented in Figure 3. At the initial stages of the aggregation, as shown by the inset of Figure 3a, λ_l_, λ_2_, and λ_3_ are almost equal, indicating the formation of small, separated spherical aggregates (Figure 3b, aggregate A). These small micelles rapidly find each other and form small aggregates with multiple cores (aggregate B). The aggregation continues by formation of anisotropic supramolecular structures and a transition from spherical particle to worm-like multicore assemblies, as shown by λ_l_ increasing, while λ_2_ and λ_3_ remain nearly constant, see aggregates C–E in Figure 3b. The shapes of these worm-like aggregates fluctuate, see aggregates D, E, and finally, the system approaches (aggregate G) and reaches (aggregate H) a final spherical structure. Deviation of the principal component values here corresponds to structural fluctuations, and the overall structure is a sphere. 

Figure 3a also presents an inset showing the corresponding asphericity, δ. In the beginning of the assembly, δ is close to unity, which matches the corresponding morphologies in Figure 3b. The final equilibrated aggregate is spherical, as shown by δ smaller than 0.01. 

To study the range of conditions where the observed multicore micellization occurs and to map out its polymer species’ sensitivity, we varied the solvent selectivity for the solvophobic and solvophilic homopolymers. This was performed by simply changing the corresponding bead–solvent interaction parameters between 35 and 115 for *a*_AW_ and between 30 and 115 for *a*_SW,_ respectively. 

**Figure 4:**
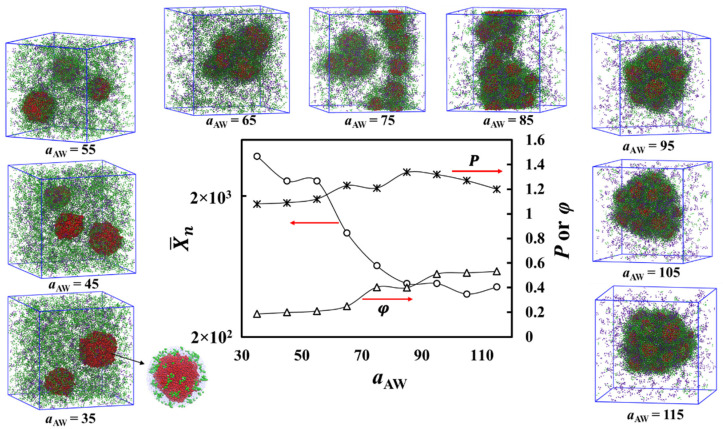
Simulation snapshots and analysis of the aggregate structural changes when a_AW_ is varied in the system containing A_19_ (4%), A_1_-*b*-S_6_ (13%), and S_6_ (3%). Solvent beads are omitted for clarity. The color scheme is the same as that of Figure 1. The data graphs show mean aggregation number ${\bar{X}}_{n}$, polydispersity index (P) of the solvophobic cores, and the order parameter (φ) of the overall aggregate versus the more solvophobic polymer–solvent interaction parameter *a*_AW_. Error bars are smaller than the symbol size. The scatter in the data is due to finite size effects in the simulations.

We first discuss the results shown in Figure 4 when the solvophobic homopolymer solvent selectivity varies by reducing the interaction between beads A and W, i.e., *a*_AW_. Decreasing *a*_AW_ from the reference value of 115 (making the bead type A less solvophobic) decreases the number of solvophobic cores. This is a direct consequence of the solvophobic polymer becoming less solvophobic or more soluble. Multicore micellization still persists for a_AW_, that is about 65. For *a*_AW_ less than that, there is a gradual transition to a regime where single-core aggregates surrounded by the solvophilic polymer form. These single-core assemblies are structurally similar to the small micelles formed in multicore aggregate formation step I, see Figure 4, visualization of a system corresponding to *a*_AW_ = 55. On the other hand, when the homopolymers become more and more soluble, the system no longer forms clear aggregates with internal structure. Instead, the polymer chains form partially soluble spherical particles in the solvent due to their attractive interactions, see Figure 4, visualization of a system corresponding to *a*_AW_ = 35. 

The mean aggregation number, ${\bar{X}}_{n}$, and polydispersity, *P*, of the solvophobic cores versus the more solvophilic polymer–solvent interaction parameter *a*_AW_ are presented in Figure 4. The data show the general trend of a decreasing *a*_AW_, leading to a significant increase in ${\bar{X}}_{n}$. There are two sharp steps in the aggregation number vs. *a*_AW_. The increase taking place with *a*_AW_ between 75 and 65 corresponds to a transition from multicore to single-core structures. The second transition from single-core aggregate to soluble particle takes place with *a*_AW_ decreasing from 45 to 35. It occurs because in this range of *a*_AW_, the solvophobic A_19_ chains are relatively solvophilic, but still assemble as spherical particles that have an interface with the solvent. This results from the stronger like–like interaction between the A beads (*a*_AA_ = 25) than between the A–W beads (*a*_AW_ = 35). Block copolymers are mostly solvated, with only a few copolymer chains remaining connected to the particle surfaces by their A block head, see Figure 4, panel corresponding to *a*_AW_ = 35 for visualization.

We note that the polydispersity index of the aggregates is relatively insensitive to *a*_AW_. Nevertheless, the snapshots in Figure 4 indicate that decreasing *a*_AW_ eventually leads to less polydisperse cores when the multicore structures are replaced by single-core aggregates. Furthermore, the variation of *a*_AW_, i.e., the solvophobicity of the solvophobic polymer, provides a means to tune the solvophobic core size for single-core aggregates. 

Additionally, the dependence of the aggregate order parameter on *a*_AW_ is shown in Figure 4. The data show a decreasing trend with decreasing *a*_AW_. This is a direct consequence of the increasing compatibility between the solvophobic and solvent beads, which leads to a smaller degree of molecular separation.

**Figure 5:**
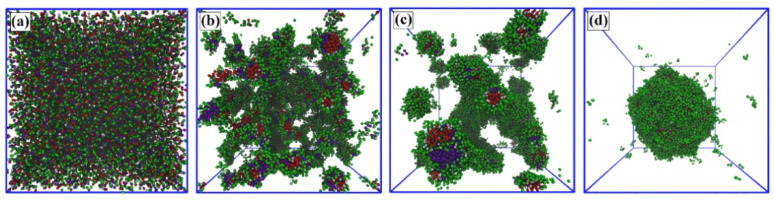
Aggregation of a system containing A_19_ (4%), A_1_-*b*-S_6_ (13%), and S_6_ (3%) in a simulation system of size (40*R*_c_)^3^, with *a*_sw_ = 65. The snapshots correspond to simulation times (**a**) initial state, (**b**) 1 × 10^3^, (**c**) 1 × 10^4^, and (**d**) 6.0 × 10^5^ steps. The color scheme follows that of Figure 1.

The second important parameter in the system is the solvophilic homopolymer solvophilicity parameter *a*_SW_, whose reference value was set to 30. Figure 5 shows simulation snapshots with a larger value of a_SW_ = 65. Increasing the solvophobicity of the S_6_ homopolymer leads to the chains entering the aggregate to reduce their solvent contact, which promotes the formation of multicore–multicompartment assemblies. Similar to the case where *a*_SW_ = 30, the more solvophobic A_19_ polymer aggregates to form cores, but now also the less solvophobic polymer S_6_ is sufficiently solvophobic to aggregate and form cores instead of just filling up the aggregate. Both types of cores are covered by the A_1_-*b*-S_6_ di-block copolymer to form small micelles. 

**Figure 6:**
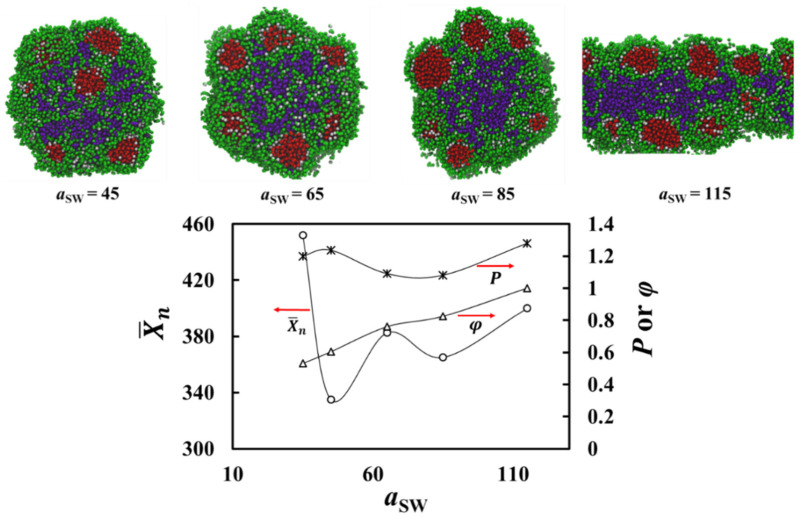
Aggregate snapshots and analysis of the aggregate structural changes when a_SW_ is varied. At the top, the cross-sections of the final morphology of the system containing A_19_ (4%), A_1_-*b*-S_6_ (13%), and S_6_ (3%) are presented. Solvent beads are omitted in the visualizations for clarity. The color scheme is the same as in Figure 1. At the bottom, the mean aggregation number, ${\bar{X}}_{n}$, polydispersity index (P) of solvophobic cores, and order parameter (φ) of the aggregates are shown as a function of the interaction parameter between the more solvophilic polymer and solvent *a*_SW_**.** Error bars are smaller than the symbol size. The scatter in the data is due to finite size effects in the simulations.

Figure 6 shows the effect of varying a_SW_ on the aggregate structure via snapshots and analysis of the aggregate structural changes as the function of *a*_SW_. The snapshots show that increasing *a*_SW_ promotes the aggregation of the solvophobic irregular cores to the center of the total aggregate and formation of a more united core. It also leads to the initial micelles having more elongated core structures. This elongation is promoted by the increasing surface contact area between the cores and the solvophilic blocks. Similar to the multicore aggregate formation, small micelles assemble together, but as there are now two types of cores, the aggregate structure is multicore–multicompartment. Notably, the larger *a*_SW_ values lead to the solvophilic S_6_ chains being present also in the center of the aggregate, surrounded by the solvophobic cores.

Furthermore, Figure 6 presents ${\bar{X}}_{n}$ and polydispersity of the solvophobic cores vs. *a*_SW_. The data show that the aggregation number does not have a simple relation to increasing *a*_SW_. While relatively large aggregates form for small *a*_SW_, a modest increase leads to a significant decrease in aggregate size, and further increasing *a*_SW_ shows that the aggregate size increases. This can be understood if one considers that a small value of *a*_SW_ = 30, i.e., good solvent solubility of the more solvophilic S_6_ polymer, leads to a minority of them assembling in the aggregate. Increasing *a*_SW_ makes the solvophilic polymer less soluble in the solvent and thus more of it assembles in the center of the aggregate. This may lead to promoting the merging of the solvophobic cores. This is shown as the increase in$\;{\bar{X}}_{n}$ in Figure 6.

The polydispersity index of the aggregates in Figure 6 remains relatively insensitive to variations of *a*_SW_. For the larger *a*_SW_ values, the increase in the polydispersity index results from the solvophilic homopolymers that remain incompatible with the solvophobic homopolymer aggregating into the aggregate. The presence of the solvophilic polymer in the aggregate imposes constraints, i.e., barriers for solvophobic beads to move in the system randomly and to find other solvophobic beads for favorable like–like interactions. This leads to more variation in the aggregate size. 

The *a*_SW_ dependence of the aggregate order parameter curve presented in Figure 6 shows an increasing trend for the order parameter with increasing *a*_SW_, i.e., increasing solvophobicity of the solvophilic polymer. This is a direct consequence of the decreasing compatibility between the solvophilic beads, solvophilic homopolymer, and solvent beads, which promotes phase separation.

### Theoretical Arguments

Formation of micelles by amphiphilic di-block copolymers in aqueous solutions is well-understood [63,64,65]. For example, for amphiphilic macromolecules in water, micellization at low temperatures is driven by a reduction in free energy by the entropy associated with the water molecules. In general, the total Gibbs free energy can be written as a sum of all the chemical potential energy terms in the solution, including those of the solvent, singly dispersed di-block copolymer, and aggregates of different sizes. In the simplest picture, micellization is driven by the free energy change, $\Delta G_{g}$, to transfer a single amphiphilic di-block copolymer molecule from the solvent to an aggregate of size *g*:

(8) $$k_{B}T\Delta G_{g}=\Delta \mu_{g}=\Delta \mu_{g,\mathrm{SO}}+\Delta \mu_{g,I}+\Delta \mu_{g,\mathrm{SI}}$$

where $\Delta \mu_{g,\mathrm{SO}}\;$is a negative contribution arising from removing the solvophobic polymer segments from having contact with the solvent (including the intramolecular/intermolecular solvophobic–solvophobic interactions), $\Delta \mu_{g,I}$ is a (positive) interface contribution, and $\Delta \mu_{g,\mathrm{SI}}$ is a (negative) contribution from the intramolecular/intermolecular solvophilic–solvophilic interactions. We note that in this model, the interface free energy contribution comes from the change in free energy per unit area when the aggregate molecules are transferred from their bulk liquid phase to either a core–corona interface or an interface with the solvent.

In contrast to the simple core-shell aggregate formation in single species di-block copolymer systems, multicore and multicompartment aggregation that emerges in multicomponent systems, such as that considered in the present DPD simulations, does not have a solid theoretical underpinning. At the microscopic level, the problem stems from the appearance of multiple competing length scales due to the many different interactions in the system. Although general formulation is lacking, the case of amphiphilic ABC copolymers has been treated with self-consistent field theory [34]. It allows one to write the total free energy as a sum of Flory–Huggins-type internal energy contribution and configurational entropy terms. In such systems, multicore assemblies can appear with a range of interaction parameters and chain lengths. 

Within the thermodynamic approach, for multicore micellization, we can write the total free energy of the multicomponent system, see Equation (8), as:

(9) $$\Delta G_{g,\mathrm{tot}}=\underset{i}{\sum }\Delta G_{g}\left(i\right)$$

where the sum over *i* is over all the molecular species (solvophobic/solvophilic homopolymers and di-block copolymer) with aggregation numbers g. Detailed evaluation of the role of various configurational and entropic contributions to $\Delta G_{g,\mathrm{tot}}$ is complicated and outside the scope of the present work. Instead, we will focus on the role of the various dominant terms from the non-bonded interactions in Equation (9) between the different species to qualitatively explain the DPD simulation data. 

**Figure 7:**
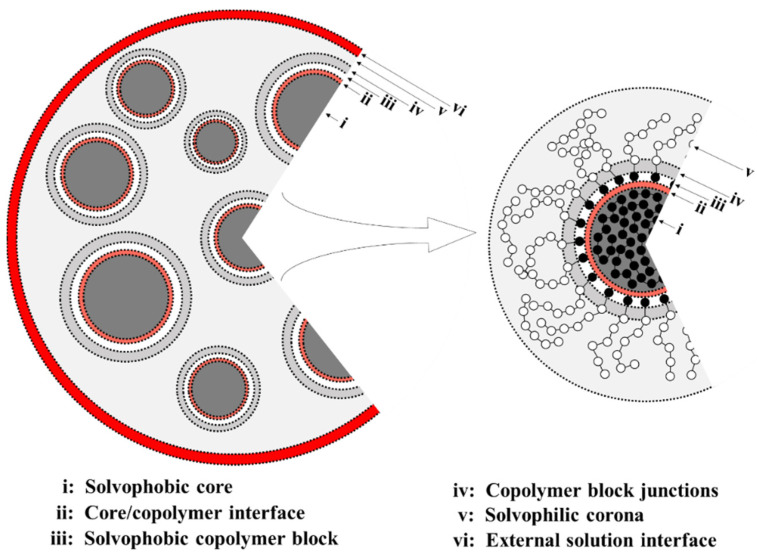
Schematic of the multicore aggregate assembled from small micelles (left panel) and the structure of one of them (right panel). In the small micelle, the black and white spheres represent solvophobic and solvophilic beads, respectively.

To this end, we focus on the multicore aggregate case and divide it into six parts, as schematically shown in Figure 7. The multicore aggregates, such as those formed in this work, are composed of the following regions:

- (i) Cores filled with solvophobic-free homopolymer A_19_ with a negative free energy contribution, $\Delta \mu_{g,\mathrm{SO}}\left(A_{19}\right),$ which dominates here, favoring aggregation of A_19_ into the core.
- (ii)–(iii) An interface between the core surface and the solvophobic A blocks of the A_1_-*b*-S_6_ copolymers that surround the cores. Here, the free energy has a positive contribution, $\Delta \mu_{g,I}\;\left(A-W\right),$ for the interface between the solvophobic core and the solvent, and a negative contribution, $\Delta \mu_{g,\mathrm{SO}}\left(A\right)$, resulting from removing core-solvent contacts by the A_1_-*b*-S_6_ copolymers.
- (iv)–(v) A corona composed mainly of the solvophilic copolymer blocks and the S_6_ homopolymer. The free energy contribution is negative and comprises of $\Delta \mu_{g,\mathrm{SI}}\left(A_{1}-b-S_{6}\right)$ and $\Delta \mu_{g,\mathrm{SI}}\left(S_{6}\right)$. This allows the solvophilic S beads to form the large corona.
- (vi) The external interface between the aggregate and solution mainly composed of solvophilic homopolymers with interface energy $\Delta \mu_{g,I}\left(S-W\right).$

First, we will qualitatively describe the multicore aggregate formation, as seen in the DPD simulations data. The multicore assemblies form because of the dominance of the term $\Delta \mu_{g,\mathrm{SO}}\left(A_{19}\right)$, resulting from the significant hydrophobicity of the A beads in A_19_. This leads to the A_19_ forming the inner core (i) in Figure 7. Following this, due to the energy cost, $\Delta \mu_{g,I}\left(A–W\right)$, from the interaction between the hydrophobic A beads and the solvent W, the A_1_-*b*-S_6_ assemble on the surface of the core to shield the cores. The copolymer assembly on the core surface results in the formation of one layer of A_1_ blocks on the core surface. This forms the interface region (ii), (iii) due to the negative contribution from $\Delta \mu_{g,\mathrm{SO}}\left(A\right)$. The S_6_ blocks and S_6_ homopolymer chains thus extend outwards, forming the solvophilic corona (iv), (v) and the small micelles. Eventually, the repulsion between S and W (S–S interaction is stronger than S–W) drives the small micelles to merge because of the large negative $\Delta \mu_{g,\mathrm{SI}}\left(S\right)$. This finally drives the system to self-assemble to a stable multicore aggregate with an interface with the solvent (vi).

Next, we consider the influence of changing the solvophobicity interaction. By decreasing *a*_AW_, the solvophobe–solvent interfacial tension decreases, and the assemblies can grow in size, see Figure 5. The multicore aggregate structures are still observed for *a*_AW_ between 115 and 65. When the A–W repulsion becomes weak enough, the A_19_ cores shielded by the copolymer become unstable as the (positive) surface energy term $\Delta \mu_{g,I}\left(A–W\right)$ (not shown in Figure 7) decreases and destabilizes the multicore configurations. This leads to the formation of single-core assemblies of one core of A_19_ and a double-layer corona made first by A_1_ and second by S_6_ blocks of A_1_-*b*-S_6_ (see Figure 4) or soluble particles of A_19_ with a sparse coating of A_1_-*b*-S_6_ (see Figure 4, configurations corresponding to *a*_AW_ = 55 and 35).

Finally, we consider the case where the solvophobicity of the homopolymer S_6_ is increased by increasing the parameter *a*_SW_ from its reference value of 30 (data of Figure 6). As expected, increasing *a*_SW_ leads to aggregation of the S_6_ chains in the emerging aggregates that now contain cores made of both the S_6_ and A_19_ molecules, as the large repulsive *a*_SW_ prevents them from mixing within the aggregates. The negative contributions from both $\Delta \mu_{g,\mathrm{SO}}\left(A_{19}\right)$ and $\Delta \mu_{g,\mathrm{SI}}\left(S_{6}\right)$ are responsible for the formation of two types of solvophobic cores. Due to the large positive contributions, $\Delta \mu_{g,I}\left(A–W\right)$ and $\Delta \mu_{g,I}\left(S–W\right),\;$the cores of A_19_ and S_6_ are shielded by the copolymer via blocks A_1_ and S_6_, respectively. The former leads to small-core–double-shell aggregates, while only irregularly shaped aggregates of S_6_ appear due to the short length of the A_1_ blocks. Changing the di-block copolymer block length ratio and increasing its degree of polymerization, the cores of S_6_ can be stabilized through small-core–double-shell aggregate formation in the overall multicore–multicompartment assembly. 

## 4. Conclusions

This work has investigated self-assembly in a three-component mixture of linear polymers composed of a solvophobic and a solvophilic homopolymer species, and an amphiphilic block copolymer species in solvent using DPD simulations. The study conditions considered fixed chain length and concentration. We showed that the linear three-component mixture self-assembles to multicore and multicore–multicompartment aggregates, in addition to standard core-shell particles. The assembly response and transitions elucidated the role of mutually competing interactions when the interactions in terms of the solvent selectivity of the components were varied. As our main result, we found that the solvophobic homopolymer assembled to finite size aggregates, that due to their high interfacial tension with the solvent, were covered and stabilized by the copolymer chains and the solvophilic homopolymer, leading to small core-shell micelles. Depending on the solvophobic polymer solvent selectivity, the small micelles either aggregate further to form multicore assemblies or fuse to single-core aggregates (in finite size, standard core-shell polymer micelles). By variation of the polymer–solvent interaction for both the solvophobic and solvophilic polymer segments, we mapped the range over which multicore aggregates assemble. Finally, we discussed the conditions for the formation and stability of multicore–multicompartment aggregates and presented qualitative free-energy arguments governing the formation of such assemblies.

The work revealed some important practical guidelines for multicore assembly formation in the present system. The main requirements are a sufficient solvophobicity of the solvophobic component and a relatively high solvophobicity difference between the solvophilic and solvophobic polymer components. Additionally, there should be two different types of immiscible solvophobic homopolymers and commensurability of each block of the copolymer with one of the homopolymers. Furthermore, decreasing the solvophobicity of the solvophobic polymer segments leads to two transitions in the aggregate structures: first, the multicore aggregates transition to phase separation of the solvophobic and the solvophilic polymer segments (single-core assemblies), and then into mixing of the components (partially soluble particles). We also demonstrated that increasing the solvophobicity of the solvophilic homopolymer changes the aggregate structure from multicore to multicore–multicompartment assemblies. In particular, in our model system, spherical multicore assemblies form for *a*_AW_ above 95 and multicore–multicompartment assemblies should have *a*_SW_ larger than 45.

The structural transitions presented here, and their relation to direct polymer chemistries via the interaction parameters, enable extracting guidelines for the multicore and the multicore–multicompartment assembly. Naturally, for real polymeric systems, practical factors such as polydispersity, the effect of solution impurities, and temperature need to be considered. However, the presented study provides a strong guideline for directing practical polymer system realizations for multicore and multicore–multicompartment assemblies by revealing trends and control features driving the assembly morphologies and their transitions.

## Figures and Tables

**Table 1:** DPD repulsive interaction parameters, *a_ij_*, between bead pairs in ${\vec{F}}_{ij}^{C}$.

i/j	A	S	W
A	25	72	115
S	72	25	30
W	115	30	25

## Data Availability

The data presented in this study are available on request from the corresponding author.

## Supplementary Materials

The following are available online at https://www.mdpi.com/article/10.3390/polym13132193/s1, Figure S1: Time evolution of the aggregate formation, Figure S2: Final simulation configuration from the simulation system (100*R*_c_)^3^ in size, Figure S3: Time evolution of the dimensionless order parameter φ, Figure S4: Time evolution of the polydispersity index versus time, Table S1: Morphological characteristics of the aggregates formed in the (100*R*_c_)^3^ simulation system.
