# Supplementary material for: Multicore Assemblies from Three-Component Linear Homo-Copolymer Systems: A Coarse-Grained Modeling Study
Source: Polymers (Basel). 2021 Jun 30;13(13):2193. doi: 10.3390/polym13132193 (PMC8272115; doi:10.3390/polym13132193)
Supplement: Supplementary file 1 [file polymers-13-02193-s001.zip › 2-JavanNikkhah_SMPolymers2021.pdf]

polymers

MDPI

Supplementary Materials

Multicore Assemblies from Three-component Linear Homo-Copolymer Systems: A Coarse-Grained Modeling Study

Sousa Javan Nikkhah[1,2]*, Elsi Turunen[3], Anneli Lepo[3], Tapio Ala-Nissila[4,5] and Maria Sammalkorpi[1,6]*

1 Department of Chemistry and Materials Science, School of Chemical Engineering, Aalto University, P.O. Box 16100, FI-00076 Aalto, Finland.
2 Department of Physics, Bernal Institute, University of Limerick, Limerick V94T9PX, Ireland; sousa.javan-nikkhah@ul.ie.
3 R&D and Technology, Kemira Oyj, P.O. Box 44, FI-02271, Espoo, Finland; elsi.turunen@kemira.com, E.T., anneli.lepo@kemira.com, A.L.
4 QTF Centre of Excellence, Department of Applied Physics, Aalto University, FI-00076 Aalto, Finland; tapio.ala-nissila@aalto.fi.
5 Centre for Interdisciplinary Mathematical Modeling and Department of Mathematical Sciences, Loughborough University, Loughborough, Leicestershire LE11 3TU, United Kingdom.
6 Department of Bioproducts and Biosystems, School of Chemical Engineering, Aalto University, P.O. Box 16100, FI-00076 Aalto, Finland; maria.sammalkorpi@aalto.fi.

* Correspondence: sousa.javannikkhah@ul.ie (S.J.N), maria.sammalkorpi@aalto.fi (M.S.)

The supplementary material consists of the following content. For the A_{19} (4%), A_1-*b*-S_6 (13%) and S_6 (3%) polymer system, we present snapshots showing aggregate structure, order parameter, and polydispersity time evolution. Simulation snapshots and morphology analysis of the larger $100R_c \times 100R_c \times 100R_c$ simulation system are also included.

Aggregate structure time evolution. The snapshots of the self-assembly process and the final morphology of the solvophobic cores for the A_{19} (4%), A_1-*b*-S_6 (13%) and S_6 (3%) system at 20% polymer concentration are presented in Fig. S1.

Examination of self-assembly response in a larger system. The self-assembly response of the same polymer composition and concentration, i.e. A_{19} (4%), A_1-*b*-S_6 (13%) and S_6 (3%), but in a larger simulation box $100R_c \times 100R_c \times 100R_c$ in size was also examined. As shown by the snapshots in Fig. S2, the aggregate morphologies obtained are highly similar to those of the smaller box. In particular, multicore aggregates similar to those in the smaller simulation system size also form in the larger system. The calculated polydispersity P, order parameter φ, and asphericity δ of the cores and overall aggregate for larger system are presented in Table S1. The data are in line with those calculated for the smaller system.

Order parameter time evolution. The time evolution of the order parameter

$$\varphi = \left(\sum_{i=1}^{N} |\varphi_{Ai} - \varphi_{Bi}|\right)/N, \quad \text{(S1)}$$

is presented Fig. S3. In this, the summation is over N slices of equal thickness along the xy-plane and in each slice the volume fractions of beads A and B are φ_{Ai} and φ_{Bi}, respectively. In Fig. S3, the embedded figure shows a magnification of the curves. It shows that the order parameter of the solvophobic cores increases first with an almost constant slope and then plateaus. This response coincides with stage (I), small micelle formation, see Fig. 2 of the main manuscript. When calculated for the entire assemblies, the order parameter time evolution shows an increase with three different slopes and then a plateau. These correspond to the stages (I), (II) and (III) described in the main manuscript. The initial slope of stage (I) follows closely the solvophobic core curve, which indicates that the dominant initial phase separation process is the solvophobic core formation. The coalescence of small multi-core aggregates in stage II shows as a steeper slope. The following decrease in slope matches with stage (III) and shows the structural change and equilibration of the final multi-core aggregate formed.

Polydispersity index. As described in the main manuscript methodology, polydispersity was assessed via the polydispersity index

$$\boldsymbol{P} = \frac{\bar{X}_w}{\bar{X}_n}, \tag{S2}$$

In this, the average particle number ($\bar{\boldsymbol{X}}_{\boldsymbol{n}}$) and the weight average ($\bar{\boldsymbol{X}}_{\boldsymbol{w}}$) are

$$\bar{\boldsymbol{X}}_{\boldsymbol{n}} = \frac{\sum_i X_i N_i}{\sum N_i} \tag{S3}$$

$$\bar{\boldsymbol{X}}_{\boldsymbol{w}} = \frac{\sum_i X_i^2 N_i}{\sum_i X_i N_i} \tag{S4}$$

The polydispersity index time evolution data are presented in Fig. S4. At the beginning of aggregation, the cores are polydisperse in size. As micellization proceeds, both the small micelles and the assembling multicore aggregate decrease in polydispersity. For the small micelle cores, the polydispersity index equilibrates to $P = 1.2 \pm 0.0002$ which corresponds to an almost monodisperse aggregate size. The equilibration takes place within 5×10^4 simulation steps for the small micelle cores and within 10^5 simulation steps for the overall aggregate. The observed independence of the polydispersity index on simulation time indicates that the cores and overall structures can be considered equilibrated in terms of their structure.

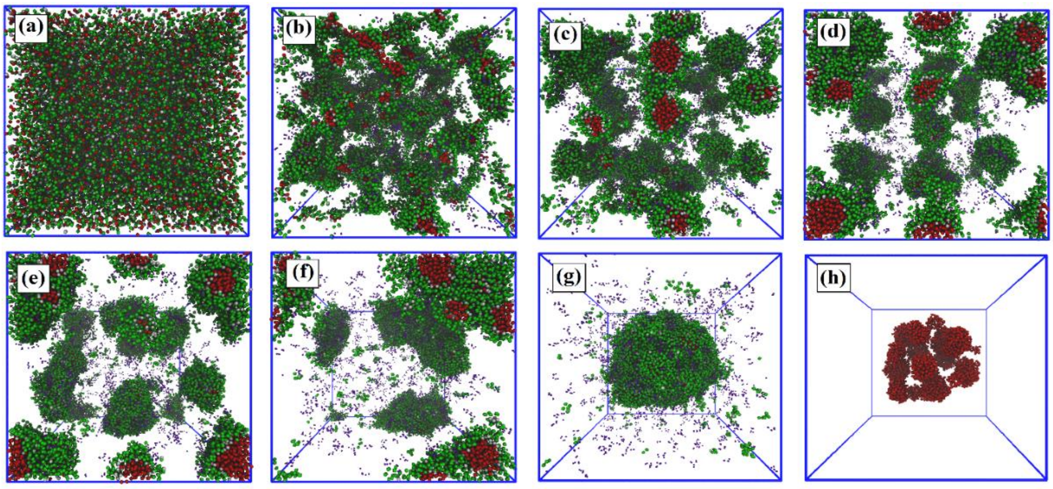

Figure S1. Time evolution of the aggregate formation in the system consisting of A_{19} (4%), A_1-*b*-S_6 (13%) and S_6 (3%) in a simulation system of size $(40R_c)^3$ and the final assembly morphology. The snapshots correspond to (a) initial state at 0 steps, (b) 10^3 steps, (c) 4×10^3 steps, (d) 2.7×10^4 steps, (e) 5.0×10^4 steps, (f) 1.3×10^5 steps, (g) 5.0×10^5, and (h) the solvophobic cores at 5.0×10^5 steps. The final equilibrium structure is a spherical multicore aggregate, (g) and (h). The color coding follows Fig. 1 of the main manuscript, i.e. solvophobic homopolymers A_{19} in red, solvophobic copolymer blocks A_1 in white, solvophilic blocks S_6 in green, and solvophilic homopolymer S_6 in purple. The solvent W is omitted in the visualization for clarity.

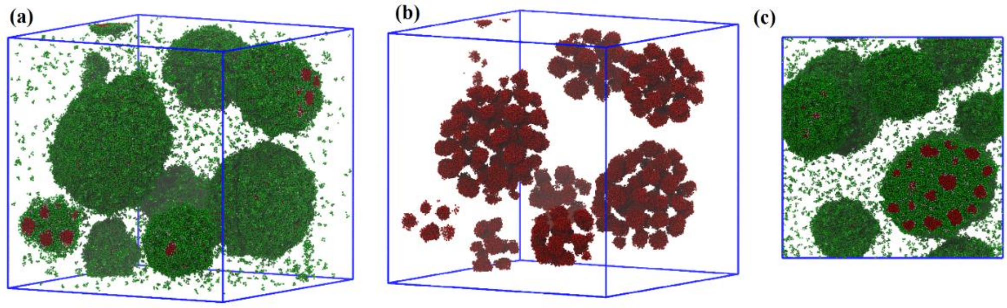

Figure S2. Final simulation configuration from the simulation system $(100R_c)^3$ in size for the A_{19} (4%), A_1-*b*-S_6 (13%) and S_6 (3%) polymer system. Subfigure (a) presents the overall picture, (b) the solvophobic cores and (c) the cross section of one multicore aggregate. The color scheme follows Fig. S1.

Table S1. Morphological characteristics of the aggregates formed in the $(100R_c)^3$ simulation system.

	Polydispersity index P	Order parameter φ	Asphericity δ
Solvophobic core	1.12 ± 0.01	0.189 ± 0.001	0.02 ± 0.04
Overall aggregate	1.82 ± 0.01	0.634 ± 0.002	0.0024 ± 0.0003

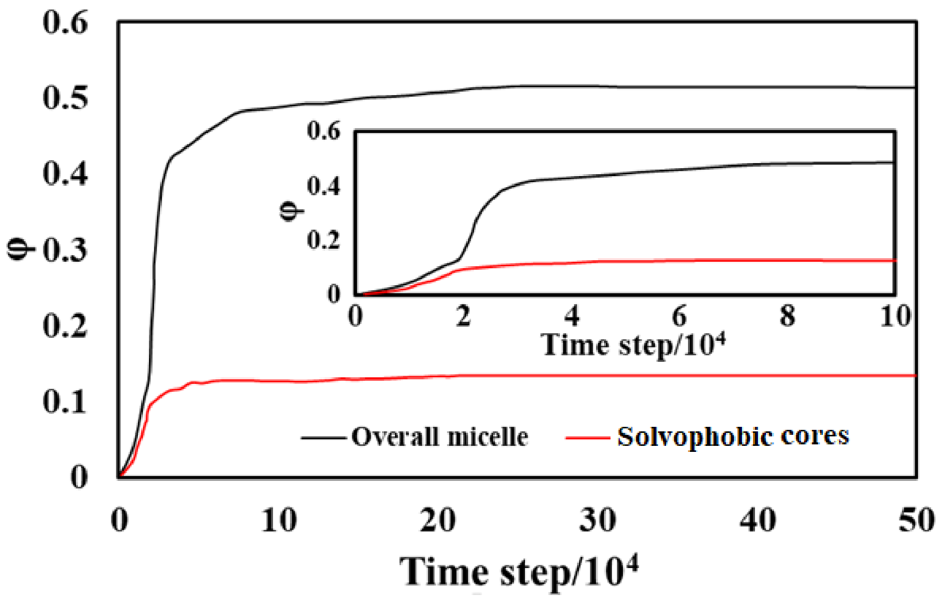

Figure S3. Time evolution of the dimensionless order parameter φ for both overall aggregate beads and solvophobic cores beads. The embedded figure shows a magnification of the first 10^4 simulation time steps.

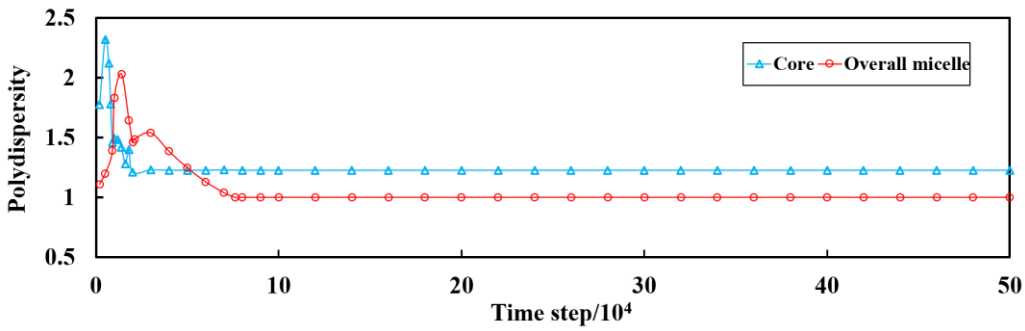

Figure S4. Time evolution of the polydispersity index versus time in a system consisting of A_{19} (4t%), A_1-*b*-S_6 (13%) and S_6 (3%). The simulation system size is $(40R_c)^3$.
